# The Moderating Role of Sensory Processing Sensitivity in Social Skills Enhancement and Bullying Prevention Among Adolescents

**DOI:** 10.3390/bs15101344

**Published:** 2025-10-01

**Authors:** Bianca P. Acevedo, Alessandra Sperati, Christopher Williams, Kenneth W. Griffin, Atena Tork, Gilbert J. Botvin

**Affiliations:** 1 Department of Psychological and Brain Sciences, University of California Santa Barbara, Santa Barbara, CA 93106, USA; 2 Department of Neurosciences, Imaging and Clinical Sciences, University G. d’Annunzio, 66100 Chieti-Pescara, Italy; alessandra.sperati@unich.it; 3 National Health Promotion Associates, White Plains, NY 10604, USA; cwilliams@nhpamail.com (C.W.); gjbotvin@med.cornell.edu (G.J.B.); 4 Department of Psychology, State University of New York at Purchase College, Purchase, NY 10604, USA; 5 Department of Global and Community Health, George Mason University, Fairfax, VA 22030, USA; kgriff4@gmu.edu; 6 Department of Biological Sciences, California State University, Sacramento, CA 95819, USA; atenatork@csus.edu; 7 Department of Population Health Sciences, Weill Cornell Medical College, New York, NY 10065, USA

**Keywords:** sensory processing sensitivity, differential susceptibility, environmental sensitivity, adolescence, social skills, cognitive skills, bullying, youth

## Abstract

Bullying is a global issue that is associated with negative life outcomes. Anti-bullying programs have been shown to be effective, but with heterogeneity across studies. Thus, we examined how sensory processing sensitivity (SPS)—a biologically based trait associated with Differential Susceptibility to environmental factors—moderates the effects of a school-based, anti-bullying program. Students (301 middle-school students, M age = 12 years) in the United States underwent a 4-week anti-bullying and competency-enhancing program. They also completed competency (e.g., social skills) and bullying prevention skills measures prior to (T1) and after the intervention (T2); and the Highly Sensitive Child Scale (measure of SPS). Results of multivariate analyses revealed that youth with higher SPS showed greater increases in decision-making, media resistance, social, and bullying prevention skills at T2. Consistent with theories of Differential Susceptibility and Environmental Sensitivity, results revealed that high SPS was associated with stronger responsivity to a psychoeducational intervention, as shown by increased cognitive, social, and behavioral domain scores. Findings from the present study underscore the moderating role of SPS on factors that impact human health and development.

## 1. Introduction

Bullying is a form of interpersonal aggression that involves persistent aggressive or abusive behavior directed toward others who are perceived to be weaker, smaller, younger, or at a relative disadvantage (APA, n.d.). Bullying can take many forms and may involve multiple individuals, including victims, bystanders, reinforcers, defenders, and bullies. Although bullying can occur in a variety of contexts, school-related bullying is most prevalent during the middle school years (Wang et al., 2009). Also, bullying is a major global public health issue, as it is associated with a variety of negative health and psychological outcomes, such as depression, anxiety, suicidal ideation, and poor academic performance (Halliday et al., 2021). Furthermore, evidence indicates that these types of negative outcomes can persist into adulthood (Arseneault, 2018).

To address this issue, there has been widespread dissemination of bullying prevention programs. Despite these efforts, recent estimates from the United States of America’s (USA’s), National Health Interview (NHI), spanning 2021–2023, suggest that there has been an increase in bullying, with approximately 33% of adolescents reporting bullying victimization in the past 12 months (Haile et al., 2024). The NHI survey also found that teenagers who had experienced bullying (vs. non-bullied teens) were about 30% more likely to report anxiety or depression in the past two weeks.

A review of anti-bullying initiatives indicates that the effectiveness of these programs is mostly mixed, with heterogeneity in effect sizes across studies (e.g., Evans et al., 2014; Gaffney et al., 2019; Yeager et al., 2015). For example, a meta-analysis of 44 studies examining the effectiveness of school-based anti-bullying programs (published between 1989 and 2009) revealed reductions in bullying of about 20–23% (Farrington & Ttofi, 2009). Another meta-analysis published between 2009 and 2016, including about 100 effect sizes from intervention programs, found reductions in bullying perpetration of about 19–20% and bullying victimization reductions of about 15–16% (Gaffney et al., 2019). The meta-analysis by Gaffney and colleagues (2019) also revealed that the effectiveness of anti-bullying programs varied substantially across studies, likely due to differences in study methods, type of intervention program, age of the sample, and the location of the study. Healy (2020) suggests that anti-bullying programs involving peer training to defend bullying victims may paradoxically result in adverse outcomes for the victims.

Studies have also examined how temperament moderates the effectiveness of anti-bullying programs. For example, one study investigated the impact of students’ temperament on the effectiveness of the KiVa anti-bullying program for early adolescents (Nocentini et al., 2019). Findings revelaed that the intervention was most effective for students with temperaments high in effortful control and medium levels of negative emotionality. Yet another temperament trait that may explain variance in bullying interventions’ outcomes is sensory processing sensitivity (SPS), a biologically based trait that is associated with depth of cognitive processing, attention to subtleties, and strong responsivity to environmental and social stimuli (Acevedo et al., 2014; Aron & Aron, 1997).

SPS (e.g., Aron & Aron, 1997)—also known as Environmental Sensitivity (Pluess, 2015) or temperamental sensitivity (Davies et al., 2021)—is based in theories of Differential Susceptibility (DS; Belsky & Pluess, 2009), Biological Sensitivity to Context (BSC; Boyce & Ellis, 2005), and Environmental Sensitivity (ES; Pluess, 2015). SPS is characterized by a low threshold of reactivity to environmental stimuli, ease of excitation, reflective processing, and differential susceptibility to both harsh and supportive environments (for review see Greven et al., 2019). While SPS has been found to be unidimensional, some studies suggest that there are three distinct groups of SPS corresponding to low, medium, and high levels of sensitivity, with high sensitivity making up about 20–35% of normative populations, as shown by studies with children and adult samples (Lionetti et al., 2018; Pluess et al., 2018).

SPS is not unique to humans as it has been observed in over 100 species (Wolf et al., 2008). In other species, it is described as a behavioral strategy that is characterized by the tendency to deliberate before acting, inhibit responses (versus acting impulsively), and engage in reflective processing. This strategy is thought to be a survival mechanism that facilitates the attainment of resources, sociality, and the avoidance of dangerous stimuli, through careful observation of the environment and comparison to past events (e.g., Acevedo et al., 2018). However, high SPS is not found in the majority of organisms within any given species because it has substantial cognitive and physiological costs (Wolf et al., 2008). In fact, a growing body of research suggests that high SPS is often associated with negative outcomes, including higher levels of depression, anxiety, and negative affect (e.g., Greven et al., 2019; Hartman et al., 2018; Pluess et al., 2023).

Conversely, in favorable conditions, high sensitivity has been shown to be associated with desirable outcomes, including positive emotions, empathy, greater self-regulatory capacity, and reward-related brain activity (Acevedo et al., 2014, 2017; for review see Greven et al., 2019; Sperati et al., 2024). Furthermore, studies examining a variety of cognitive–behavioral interventions indicate that SPS is associated with enhanced outcomes including improved cognitive function (Acevedo et al., 2023), enhanced learning and memory (Marhenke et al., 2023), and greater decreases in depression among youth (Pluess & Boniwell, 2015). For example, Pluess and Boniwell (2015) examined the effectiveness of a cognitive–behavioral intervention focused on reducing depression among pre-adolescent girls. The study found that participants with higher (vs. lower) SPS showed greater, and sustained, decreases in depression up to 12 months, post-intervention. In another study, researchers investigating the moderating influence of SPS on the efficacy of the KiVa anti-bullying program (Kärnä et al., 2011) among middle-school students in Tuscany, Italy found that higher sensitivity was associated with better treatment response to the intervention (Nocentini et al., 2018). Findings also revealed that highly sensitive boys benefited significantly more from the anti-bullying program, showing the greatest reductions in bullying victimization behaviors and internalizing symptoms, compared with less sensitive children. In summary, consistent with DS, ES, and BSC theories, a growing body of research suggests that SPS is associated with beneficial outcomes in response to positive interventions.

### Current Study

The goal of the present study was to examine the moderating impact of Sensory Processing Sensitivity (SPS) on pre- to post-test changes in skills outcomes among middle-school students participating in a bullying prevention program in the United States (USA). We predicted that higher SPS would be associated with better anti-bullying intervention outcomes, measured as increases in competency and bullying prevention skills.

## 2. Materials and Methods

### 2.1. Participants

Participants were 301 middle-school students (56% females) recruited from middle schools in the USA. The sample was derived from a larger study examining the effectiveness of a bullying prevention program (Williams et al., 2023). The mean age of participants was 11.7 years of age (SD = 0.59, range = 10 to 14 years). Forty-four percent were 6th graders and 56% were 7th graders. The sample was somewhat diverse, with most students identifying as White (66%), followed by Hispanic-Latino (15%), Black/African-American (11%), Asian (4%), Native Hawaiian or other Pacific Islander (3%), and American Indian/Alaska native (1%).

### 2.2. Procedures

A complete description of the study procedures is provided in Williams et al. (2023). The study was approved by the Institutional Review Board of National Health Promotion Associates and it was conducted in accordance with the Declaration of Helsinki. Between the 2018 and 2019 school terms, data were collected for a randomized trial of an anti-bullying intervention. Students in the control group received Life Skills Training (LST; Botvin et al., 2006; Botvin & Griffin, 2015), and those in the experimental group received LST plus enhanced anti-bullying modules. Both conditions (experimental and control) were delivered during school class sessions by regular classroom teachers. The sessions lasted approximately 45 min, and involved small group discussions, role-playing, and didactic lectures.

The universal, evidence-based LST program (Botvin et al., 2006) is organized into four components teaching students: Personal Competence, Social Competence, Substance Use Resistance, and Bullying Resistance. The enhanced anti-bullying component of the program includes (1) classroom sessions; (2) an interactive educational video game that includes content on (a) social and personal competence skills, and (b) anti-bullying norms, attitudes, and skills; and (3) e-learning modules for caregivers and school personnel to reinforce anti-bullying messages at home and school. Participating students completed online surveys prior to, and four weeks after, completing the intervention. The online surveys included questions about self-reported demographics, competency skills, and bullying prevention skills. Participants in the experimental (anti-bullying) condition also completed a scale assessing SPS. Thus, our analytic sample was limited to students in the experimental condition who completed the SPS measure, permitting us to investigate the main objectives of this study.

### 2.3. Measures

#### 2.3.1. The Highly Sensitive Child Scale

SPS was measured with the six-item Highly Sensitive Child Scale (HSC) Scale, short-form (Pluess et al., 2018). The HSC scale assesses awareness of subtleties in the environment, sensitivity to sensory stimuli, and the tendency to become overwhelmed in response to many stimuli. Sample items include: “I don’t like loud noises,” “I love nice smells,” and “I notice when small things have changed in my environment.” All questions are answered on a 7-point Likert scale (from 1 = not at all, to 7 = extremely), with higher scores indicating higher SPS. The internal consistency for the HSC scale (α = 0.77, CI = 0.73–0.81) was comparable to other studies assessing self-reported sensitivity among children and adolescents, with alpha (α’s) ranging from 0.71 to 0.79 (Lionetti et al., 2018; Pluess et al., 2018). To create HSC Scale categories, we used cut-off scores as proposed by Lionetti et al. (2018), resulting in SPS groupings as follows: Low SPS (*n* = 85, 28.5%; HSP score M = 2.8, *SD* = 1.0); Medium SPS (*n* = 145, 48.7%; HSP score M = 4.8, *SD* = 0.5); and High SPS (*n* = 68, 22.8%; HSP score M = 6.1, *SD* = 0.4).

#### 2.3.2. Skills Knowledge

The Skills Knowledge Scale (SKS; Botvin & Griffin, 2015) was used to assess changes in competency and bullying prevention skills, associated with undergoing the LST plus the anti-bullying program. The SKS includes 36 items that assessed decision-making, media resistance, coping with anxiety, coping with anger, communication, conflict resolution, bullying prevention, and social skills. All items were measured on a five-point Likert scale (1 = strongly disagree to 5 = strongly agree). The SKS has been shown to have adequate internal reliability (Macaulay et al., 2002) and high internal consistency, with α’s ranging from 0.73 to 0.89 (Epstein et al., 1997).

Decision-making skills were measured with three items: “If I need to make an important decision, I take the time to clarify the decision, consider alternatives, and choose the best option,” “When I am making an important decision, I try to get all of the information I need,” and “It’s a good idea to make a decision first and then think about the consequences later.” Media resistance skills were measured with two items: “I believe what advertisers claim about a product, without thinking much about it” and “If an ad has an expert saying that the product works, it must be true.” Social skills were measured with two items including: “I ask a question to start a conversation with someone I just met” and “When ending a conversation with someone I just met, I say that I enjoyed talking to them.”

Bullying was assessed with 11 items measuring bullying resistance skills and bystander intervention skills. Bullying resistance skills were measured with seven items, such as, “I would say no if someone tried to get me to bully another student” and “I would say no if someone tried to get me to cyberbully another student.” Bystander intervention skills were measured with four items such as, “If I saw someone being bullied, I would directly confront the person doing the bullying,” and “If I saw someone being bullied, I would cause a distraction to make it stop.”

#### 2.3.3. Data Analysis

First, we explored the frequency and distribution of missing data. As the percentage of missing values in the current sample was 6% and missing data were completely at random (as suggested by results from Little’s MCAR test (i.e., *p* = 0.99), we adopted a listwise deletion approach. We also investigated all variable distributions and potential gender differences in HSC scores by conducting an independent sample t-test and computing bivariate associations among all variables of interest. The HSC variable approached a normal distribution consistent with the literature (Figure S1). Results of an independent samples t-test (t = −2.59, df = 241.76, *p* = 0.01) showed that mean HSC scores differed significantly between females and males, with females (M = 4.69, SD = 1.23) showing higher mean HSC scores than males (M = 4.27, SD = 1.55). Thus, we controlled for gender in our analyses. All analyses were conducted using the statistical software RStudio 2023.12 (R Core Team, 2020).

We conducted a combination of bivariate and multivariate statistical analyses including t-tests, correlations, and multiple regressions. To explore the hypothesized differences in decision-making, media resistance, social, and bullying prevention skills as a function of SPS levels, we conducted a series of linear regressions adopting a model comparison approach. We first computed models considering HSC scores and each pre-intervention (T1) variable as a predictor of each outcome variable’s score at post-intervention (T2), controlling for gender. Next, we performed a series of interaction models including SPS as the moderating variable, to evaluate whether inclusion of SPS as the interaction term would improve the model’s prediction. We compared the main effect and interaction effects models using the R^2^ (i.e., the total variance of the outcome variable accounted for by the model), the AIC (Akaike, 1974) indices, the BIC (Schwarz, 1978), and the Akaike weights (Burnham & Anderson, 2002). According to AIC and BIC criteria, the lower the value, the better the model was at predicting data, while for R^2^ and Akaike weights, ranging from 0 to 1, the higher the value, the better the model was at describing data accurately (Wagenmakers & Farrell, 2004; Vandekerckhove et al., 2015; McElreath, 2018). After selecting the best fitting models, we further explored significant interaction models by plotting simple slopes in conditional plots, using SPS categories as delineated by Lionetti et al. (2018).

## 3. Results

### 3.1. Descriptive Statistics

Means and standard deviations for outcome variables are reported in Table 1. The mean HSC scale scores for the sample of adolescents was 4.5 (SD = 1.4, range: 1–7), comparable to those observed in adolescent samples in the United Kingdom (Pluess et al., 2018).

### 3.2. Bivariate Correlations

Bivariate correlations among major variables are reported in Table 2. With respect to competency and bully related skills, results showed that SPS was moderately associated with decision-making (r = 0.16, *p* < 0.05) and bullying resistance skills (r = 0.26, *p* < 0.001) at T1. When considering outcomes at T2, SPS was slightly to moderately associated with decision-making, media resistance, social, bystander intervention and bullying resistance skills with r values ranging from 0.15 to 0.25.

### 3.3. Main and Interaction Effect Models

**Figure 1:**
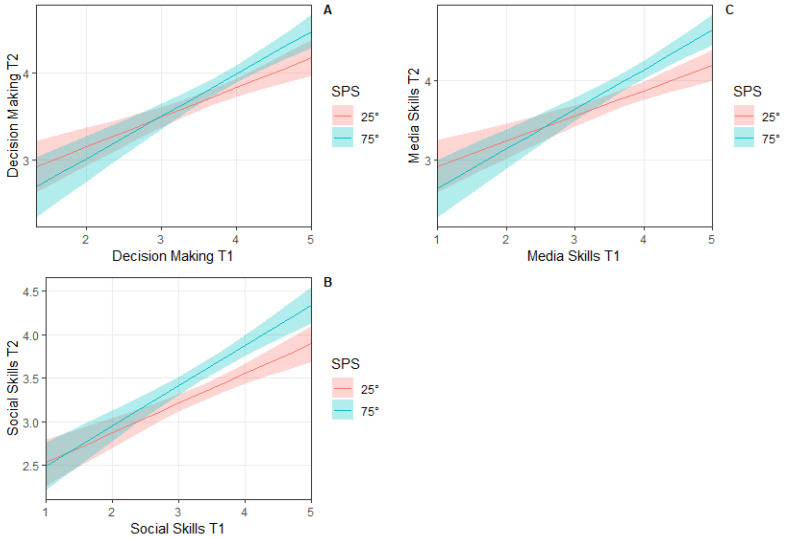
Decision-making skills (**A**), social skills (**B**), and media resistance skills (**C**) predicting at T1 (pre-intervention, x-axes), predicting decision-making skills, social skills, and media resistance skills at T2 (post-intervention, y-axes), as a function of low and high Sensory Processing Sensitivity (SPS), measured with the HSC Scale. Shaded bands represent the uncertainty of estimates (95% Confidence Interval). Note: SPS was divided into low (below the 25th quantile) and high (above the 75th quantile).

Linear regression model comparisons showed that for three out of five outcome variables at T2 (i.e., decision-making, media resistance, and social skills), the interaction effects models (i.e., variable at T1 × SPS) outperformed the main effects only models (see Table 3, Table 4 and Table 5). Specifically, SPS was significantly and positively associated with decision-making skills at T1 (β = 0.71, *p* = 0.03), media resistance skills at T1 (β = 0.83, *p* < 0.01), and social skills at T1 (β = 0.57, *p* = 0.03) in predicting the corresponding outcomes at T2. To follow-up on the significant interaction effects, we created exploratory simple slope plots for low (below the 25th percentile), medium, and high (above the 75th percentile) HSC scale scores (see Figure 1). Inspection of the plots indicated that decision-making, media resistance, and social skills at T1 were positively associated with outcomes of each variable at T2, and this association was stronger for youth with higher HSC scale scores (Figure 1). Specifically, adolescents with high SPS levels showed the largest increases in decision-making, media resistance, and social skills from pre- to post-intervention.

For bullying-related outcomes, the main effect models for bystander intervention and bullying resistance skills predicted better fit data, compared with the interaction effects models. Results showed a positive and significant effect of both SPS and the bullying-related variables at T1, in predicting bystander intervention skills at T2 (SPS = β = 0.16, *p* < 0.01; bystander intervention T1 = β = 0.32, *p* < 0.001), and bullying resistance skills at T2 (SPS = β = 0.21, *p* < 0.001; bullying resistance T1 = β = 0.35, *p* < 0.001).

## 4. Discussion

Bullying is a global issue that has significant lifetime mental health consequences for both victims and perpetrators. Thus, implementation of school-based anti-bullying programs has become more common around the globe. Studies examining the efficacy of anti-bullying programs have identified a number of individual factors, including social competence and problem-solving, that may protect youth from bullying victimization (Zych et al., 2019). Also, although anti-bullying programs have been shown to be effective by meta-analyses, no consistent pattern of findings has emerged across studies (e.g., Gaffney et al., 2019). To address this concern, some studies have started to examine the impact of factors that may account for the variability in outcomes associated with anti-bullying programs.

In the present study, we examined the moderating role of Sensory Processing Sensitivity (SPS)—a biological trait associated with depth of cognitive processing, emotional reactivity, awareness of subtleties, and greater responsivity to environmental and social stimuli (Acevedo et al., 2014; Aron & Aron, 1997)—in association with an anti-bullying intervention. Our primary objective was to determine whether SPS moderates pre- to post-test changes in key skills outcomes among students participating in a prevention program. Using outcome data from a previously evaluated anti-bullying intervention trial (Williams et al., 2023), in the current study, we examined whether SPS would moderate the programs’ outcomes on competency skills and bullying prevention skills among early adolescents. We hypothesized that youth with higher sensitivity would show greater increases in competency and bullying prevention skills, consistent with SPS’s central feature of enhanced responsivity, which is proposed to be mediated via depth of cognitive processing and emotional reactivity.

Our results revealed that higher sensitivity was associated with greater increases in decision-making, media resistance, and social skills scores among middle-school students who participated in the 4-week, school-based LST plus bullying prevention program. Also, higher SPS was associated with stronger bystander intervention and bullying resistance skills, post-intervention. These findings add to the body of literature examining factors that may influence responsiveness to bullying prevention programs, and suggest that it is important to consider individual differences in SPS among youth to identify those that may be more responsive to bullying preventative and treatment efforts.

Results of the present study also support theories suggesting that SPS is associated with Differential Susceptibility to both supportive and negative environments (e.g., Belsky & Pluess, 2009). For example, research suggests that in response to negative contexts, high SPS is associated with adverse outcomes, such as depression, anxiety, and emotional over-reactivity (e.g., see Greven et al., 2019, review). However, high SPS is also related to stronger beneficial outcomes in supportive contexts, such as when parenting is high in maternal warmth (e.g., Sperati et al., 2024), and when exposed to interventions for reducing depression (e.g., Pluess & Boniwell, 2015) and bullying-related symptoms (Nocentini et al., 2018).

Our results revealed that youth with higher SPS showed superior increases in cognitive-related competencies (e.g., decision-making and media resistance skills) and social skills in response to the LST plus anti-bullying program. Also, the sample of young adolescents with higher sensitivity showed greater increases in prosocial skills, including bullying prevention and bullying resistance skills, in comparison to their less sensitive peers. These findings are in line with theories and prior empirical studies showing that SPS is associated with greater depth of processing and attunement to social and affective cues (Aron, 2002).

For example, neuroimaging studies have shown that SPS is associated with increased activity in brain regions important for empathy, emotion, and attention (e.g., the anterior insula, inferior frontal gyrus, and cingulate) in response to positive and negative emotionally evocative images (e.g., Acevedo et al., 2018). In two separate studies examining the neural correlates of SPS in response to emotional stimuli, findings revealed that SPS was positively associated with reward-related activity in response to viewing positive images, such as an image of a loved one smiling (Acevedo et al., 2014, 2017). However, higher levels of SPS were also found to be related to diminished reward brain activity in response to viewing negative images (e.g., a dead animal); but these effects were attenuated for participants who reported having had a higher-quality childhood (Acevedo et al., 2017).

Thus, highly sensitive youth may be especially attuned to social cues of bullying, as well as anti-bullying interventions, which include emotional and social content. Also, as suggested by theory and research, high SPS youth may be more inclined to act pro-socially and carefully consider their decisions; as well as integrating the anti-bullying educational material more thoroughly. Overall, our findings suggest that youth with higher SPS show stronger responsivity to interventions focused on prosocial skills, likely due to the trait’s cardinal features of enhanced cognitive processing and attunement to social information.

### 4.1. Strengths and Limitations

This study had several methodological strengths including (a) the use of a well-established data collection protocol for school-based research; (b) self-report surveys and measures with strong psychometric properties including high reliability and construct validity; and (c) multivariate models that account for heterogeneity in the data beyond simple main effects to explore the complexity of the relationship between covariates and behavioral outcomes. Furthermore, this was the first study to examine the influence of SPS on outcomes related to LST with an enhanced anti-bullying component.

However, further research is needed on this topic with larger samples while addressing the limitations of the present study. Another limitation was the sole reliance on self-report measures. Although self-reports are commonly used in school-based research, objective measures such as performance metrics (e.g., academic performance and physiological measures), non-self-report behavioral ratings (e.g., teacher, parent, and peer ratings), and behavioral observations could improve the methodological rigor of this and related studies. Furthermore, although the larger randomized trial from which the present sample was drawn from included a control group (Williams et al., 2023), the HSC Scale was only completed by the students receiving the anti-bullying program. Thus, we were unable to conduct analyses comparing treatment and control groups in the present study. However, assessment of the effectiveness of the anti-bullying program was not an objective of the present study, as those results were already published by Williams et al. (2023). However, it is possible that when examining SPS’ effects in both the intervention and control groups, that the more sensitive students might show responsiveness to the control group also. Furthermore, we were not able to control for other covariates, such as classroom climate and teacher engagement in the present study. Future studies may seek to address these limitations.

Finally, the regression model with interaction effects revealed that SPS was a significant moderator of responses to the anti-bullying program, such that youth with higher (vs. lower) sensitivity showed significant increases in decision-making, social, media resistance, and bullying prevention skills. Interestingly, this pattern was even stronger when highly sensitive adolescents had higher scores at T1 in these skills, suggesting a greater benefit related to high sensitivity from both the program and internal strengths. These findings provide insights for enhancing future interventions, such as targeting specific skill areas and designing personalized strategies for skills enhancement, perhaps focusing on high-risk students. 

### 4.2. Future Directions

A logical extension of the present study would be to examine intervention effects between treatment and control groups (including additional covariates), and to investigate whether the effects of the experimental condition are more sustainable and stronger among youth with higher sensitivity. There is some evidence suggesting that SPS is associated with sustained benefits up to 12-months, post-treatment, among adolescents who participated in a school-based program for depression (Pluess & Boniwell, 2015). Also, as mentioned above, the methodological rigor of school-based intervention studies could be improved by assessing outcomes with measures that provide greater objectivity than self-reports, such as behavioral, observational, and parent-, peer-, or teacher-rated assessments. Furthermore, future studies could examine mental health outcomes (e.g., anxiety, depression, and internalizing symptoms) and intra and interpersonal outcomes (e.g., relationship quality, self-esteem, and peer relationship quality). Future research could also examine whether youth benefit from personalized intervention strategies. Screening participants for SPS may facilitate the use of targeted, brief, and efficient interventions that minimize burden and reduce costs (Thibodeau et al., 2016).

## 5. Conclusions

The present study showed that pre-post increases in competency and bullying prevention skills among youth who participated in an LST plus anti-bullying program were moderated by the biologically based sensory processing sensitivity (SPS) trait. Analyses revealed that highly sensitive youth showed the greatest increases in several personal and interpersonal competencies, including decision-making skills, media resistance skills, social skills, bullying resistance skills, and bullying bystander intervention skills. These results are likely mediated by cardinal features of SPS, including depth of cognitive processing and greater attunement to social/affective stimuli, as supported by prior research and SPS theory. This study provides evidence that an anti-bullying intervention was especially effective among youth with higher levels of SPS, and highlights the benefit of considering SPS when implementing and evaluating psychoeducational interventions.

## Figures and Tables

**Table 1:** Descriptive statistics for the anti-bullying program outcome variables, pre- and post-intervention.

	Pre-Intervention	Post-Intervention	
	Mean (SD)	Mean (SD)	Cohen’s d
Decision-making skills	3.7 (0.7)	3.8 (0.7)	0.08
Media resistance skills	3.8 (0.9)	3.9 (0.9)	0.13
Social skills	3.3 (0.9)	3.4 (0.8)	0.12
Bystander intervention skills	3.4 (0.8)	3.5 (0.7)	0.004
Bullying resistance skills	4.4 (1.1)	4.4 (1.0)	0.02

**Table 2:** Bivariate associations between Sensory Processing Sensitivity with competency and bullying prevention skills, pre (1) and post (2) intervention.

	1	2	3	4	5	6	7	8	9	10	11	12
1. SPS												
2. DMSk1	0.16 *											
3. MediaSk1	0.12	0.20 **										
4. SocSk1	0.00	0.31 ***	−0.17 *									
5. BystSk1	−0.03	0.22 **	−0.16 *	0.44 ***								
6. BulResSk1	0.25 ***	0.20 **	0.29 ***	0.10	0.20 **							
7. DMSk2	0.21 **	0.46 ***	0.15 *	0.15 *	0.10	0.27 ***						
8. MediaSk2	0.16 *	0.28 ***	0.50 ***	−0.08	0.01	0.25 ***	0.32 ***					
9. SocSk2	0.21 **	0.16 *	−0.08	0.45 ***	0.33 ***	0.18 **	0.31 ***	−0.12				
10. BystSk2	0.15 *	0.20 **	−0.08	0.25 ***	0.34 ***	0.23 ***	0.43 ***	−0.01	0.46 ***			
11. BulResSk2	0.23 ***	0.16 *	0.16 *	0.18 **	0.22 **	0.45 ***	0.34 ***	0.27 ***	0.34 ***	0.44 ***		
12. Gender	0.19 **	0.08	0.07	−0.02	0.04	0.16 *	0.09	0.00	0.09	0.17 *	0.15 *	
13. Age	0.00	−0.11	−0.06	−0.08	−0.04	−0.15 *	−0.06	−0.00	−0.01	0.07	0.04	−0.07

Note. SPS = Sensory Processing Sensitivity, DMSk = Decision-Making Skills, MediaSk = Media Resistance Skills, SocSk = Social Skills, BystSk = Bystander Intervention Skills, BulResSk = Bullying Resistance Skills, 1 = Pre-Intervention, 2 = Post-Intervention. * = *p* < 0.05; ** = *p* < 0.01; *** = *p* < 0.001.

**Table 3:** Comparison of regression models considering decision-making skills at T1 and SPS, in predicting decision-making skills at T2.

Models	R2	AIC	Delta	Akaike Weights	LogLik
Model 3 (SPS × decision-making at T1)	0.20	522	0.00	0.78	−255
Model 2 (SPS + decision-making at T1)	0.18	524	2.56	0.22	−257

Note. SPS = Sensory Processing Sensitivity; LogLik = log-likelihood; × = interaction.

**Table 4:** Comparison of regression models considering media skills at T1 and SPS, in predicting media skills at T2.

Models	R2	AIC	Delta	Akaike Weights	LogLik
Model 3 (SPS × media skills at T1)	0.23	629	0.00	0.96	−308
Model 2 (SPS + media skills at T1)	0.20	636	6.57	0.04	−312

Note. SPS = Sensory Processing Sensitivity; LogLik = log-likelihood, × = interaction.

**Table 5:** Comparison of regression models considering social skills at T1 and SPS, in predicting social skills at T2.

Models	R2	AIC	Delta	Akaike Weights	LogLik
Model 3 (SPS × social skills at T1)	0.28	524	0.00	0.78	−256
Model 2 (SPS + social skills at T1)	0.26	526	2.53	0.22	−259

Note. SPS = Sensory Processing Sensitivity; LogLik = log-likelihood, × = interaction.

## Data Availability

The raw data used in this study are available from the co-author Christopher Williams upon a reasonable request.

## Supplementary Materials

The following supporting information can be downloaded at: https://www.mdpi.com/article/10.3390/bs15101344/s1, Figure S1. Sensory Processing Sensitivity variable distribution.
