# Supplementary figures and images for: The Moderating Role of Sensory Processing Sensitivity in Social Skills Enhancement and Bullying Prevention Among Adolescents
Source: Behav Sci (Basel). 2025 Oct 1;15(10):1344. doi: 10.3390/bs15101344 (PMC12561777; doi:10.3390/bs15101344)

**Figure S1.** Sensory Processing Sensitivity variable distribution.

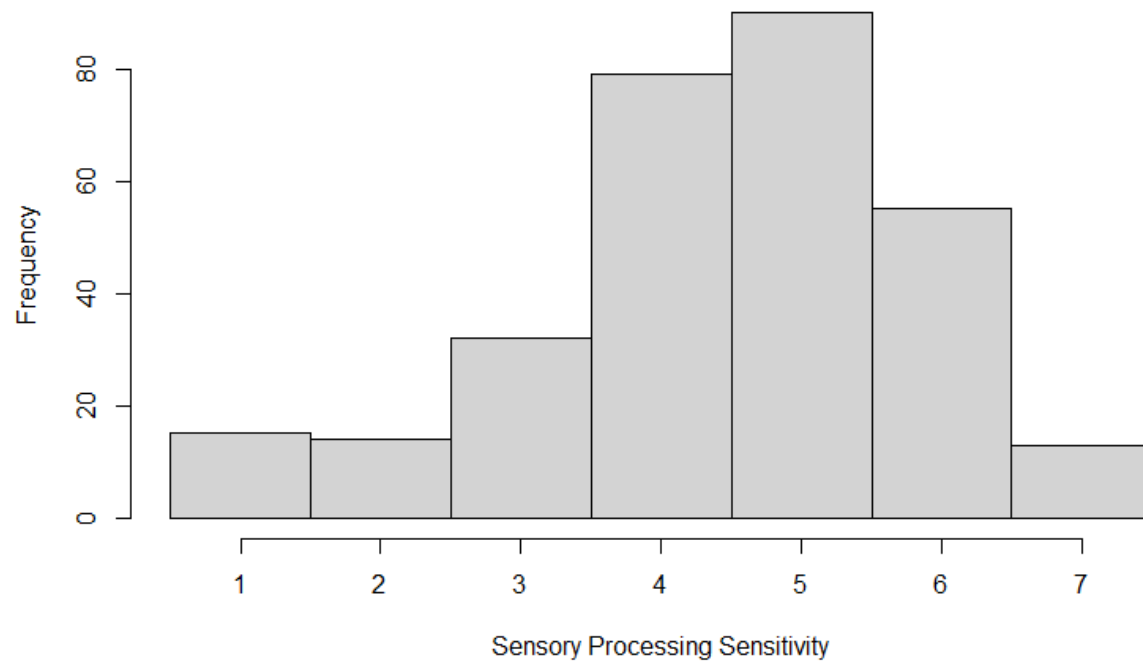

Supplement: Supplementary file 1 [file behavsci-15-01344-s001.zip › behavsci-3735546-supplementary.pdf]
